# Visual mental imagery and symptoms of depression – results from a large-scale web-based study

**DOI:** 10.1186/s12888-015-0689-1

**Published:** 2015-12-02

**Authors:** Charlotte Weßlau, Marie Cloos, Volkmar Höfling, Regina Steil

**Affiliations:** Department of Clinical Psychology and Intervention, Institute of Psychology, Goethe University Frankfurt, P.O. Box 11 19 32–120, 60054 Frankfurt Main, Germany

**Keywords:** Visual imagery, Mental images, Depression, Maintenance of depressive symptoms

## Abstract

**Background:**

Mental imagery may influence the onset and maintenance of depression, but specific mechanisms have not yet been determined.

**Methods:**

Nine hundred twelve participants completed questionnaires on positive and negative mental images, as well as images of injury and death that lead to positive emotions (“ID-images”), and depressive symptomatology. The assessment was carried out online to reduce effects of social desirability.

**Results:**

Positive images were reported by 87 % of the sample, negative images by 77 %. ID-images were present in one-third of the sample. A connection with depression severity was found for the absence of positive mental images and the presence of negative images as well as ID-images. Higher depression scores were associated with more frequent and vivid negative images, greater imagery distress, and a higher proportion of negative relative to positive images.

**Conclusions:**

Mental images are clearly related to depression. Future research should focus on ID-images and their connection to suicide-risk in depressed patients.

## Background

Negative mental images are common in clinical and non-clinical populations [1] and appear to be a transdiagnostic feature in a variety of mental disorders [2, 3]. Mental imagery can occur across a range of sensory modalities, like auditory [4] or gustatory [5], but the visual modality is the one studied most [6]. Visual mental imagery can be described as “seeing with the mind’s eye” [7], with the image being primarily visual in nature (see e.g. [8]). Negative mental images may represent intrusive recollections of unpleasant autobiographical events or non-memory based scenes, or they may portray fears of possible future disasters; a phenomenon which has been termed (involuntary) mental time travel [9]. Further, they can include intentionally produced images of future self-harm and suicidal ideation, so called “flash-forwards” [10], or intrusive forms of mental “pre-experiencing” [11]. Nonetheless, research has only recently included images of objectively negative content (e.g., suicide or violent revenge), which may lead to feelings of comfort rather than distress [10, 12], a phenomenon that we termed images of injury and death (ID-images). It has been hypothesized that while most individuals with affective disorders seem to find suicidal imagery aversive [13], some might find comfort in them and may use these images an escape strategy from emotional distress [14]. It is important to note that future directed imagery can affect the likelihood of actual behavior [15].

It has been found that mental imagery exists very early on in childhood development, however it is not yet clear if and how mental imagery can play a causal role in mental disorders [16]. Research indicates though that distressing imagery may influence the maintenance of psychopathology [17–19]. Mental images substantially impact emotions, exceeding the impact of verbal cognition [20], which could be of vital importance in emotional disorders, such as major depression (MD). Negative mental images might not only lead to emotional distress but can also facilitate the retrieval of further negative imagery, for example in the form of autobiographical memories [21]. They can exacerbate current mood states and might contribute to mood instability [22]. The focus of research has only recently begun to shift to the roles of distressing images [23] and the lack of positive imagery [24] in depression [8]. Existing studies have often been limited by small sample sizes or methodological problems; therefore, little is currently known regarding the specific mechanisms by which mental imagery contributes to psychopathology outside of the Posttraumatic Stress Disorder (PTSD). Studies so far indicate that mental imagery and associated cognitive processes, like suppression or dysfunctional appraisals, may contribute to the aggravation and maintenance of depressive symptoms [8].

Both currently depressed patients and those in remission frequently suffer from intrusive images and memories [1, 25, 26]. In depression, these visual images are often accompanied by high levels of vividness and distress [1] and they have been found to interfere with daily activities [27]. Perceived nowness of intrusive memories has been found to be predictive of both imagery distress and symptom severity in depression [28]. Contents of intrusive memories in depression are often based on critical life-events [8] and relate to topics like interpersonal problems, illness, death or injury to the patient or significant others, as well as threat or threat of assault regarding the patient [23]. Studies have also found a limited ability to generate positive future-related images in high-dysphoric individuals [24] as well as a connection between deficits in memory specificity and deficits in the specific imaginability of future related imagery in suicidal patients [29]. The recall of positive memories may repair sad mood in non-depressed individuals [30, 31]. However, this recall has no effect on formerly depressed participants and may even lead to mood deterioration in those who are currently depressed. Depressed mood may impair access to positive autobiographic memories and that even if those memories are actively recalled, their positive influence on emotions is scant [32]. In addition, depressed mood leads to enhanced retrieval of negative autobiographical memories [33].

The overall aim of this online study was to assess the connection between imagery characteristics and depressed mood and their stability or variability over time. We extended previous research by obtaining information on participants’ positive imagery and ID-images and repeatedly assessing both mental images and depressive symptoms. We developed path models regarding the prevalence of different imagery types and their influence on depressed mood. In addition, more specific models were created to measure the influence of imagery characteristics, such as frequency, nowness, vividness or controllability, on imagery distress and subsequent mood.

Based on previous research, we assessed the collective effects of all imagery types on depression score. We hypothesized that the number of negative mental images and ID-imagery would be connected to high levels of depression and, in contrast, that experiencing positive mental images would be associated with low depression scores. We further assumed that the quantity of different mental images as well an imbalance between positive and negative mental images would be connected to depression severity. To assess the specific mechanisms through which mental images are connected to self-reported depression severity, we conducted analyses on imagery characteristics, like vividness, distress/pleasantness, nowness and controllability. It was predicted that negative mental images would lead to a deterioration of mood, whereas positive mental images would lead to better mood following the image and that this relationship would be connected to depression severity. As the relationship between the characteristics of ID-images and depression has not yet been assessed in detail, we used this study as an exploratory analysis of this phenomenon. Cross-sectional as well as longitudinal path models and regression analyses were developed to assess the influence of imagery characteristics on depression scores, both at baseline and at the 8-week follow-up.

## Method

We present data from a repeated-measures, large-scale web-based study of self-selected adults aged 18 to 65. The assessment was conducted using Unipark (QuestBack GmbH, Germany), an online survey software program. Participants were asked to generate and note down a personalized code to match their data from t_1_ and t_2_ if they decided to take part at both time points. Upon approval, an email address was required and was stored separately from the rest of the data. The survey was available in German only. In addition to a questionnaire regarding mental images, we analyzed data from a questionnaire for depressive symptoms. Permission from the local ethics committee of the department of psychology and sport science (Ethikkommission Fachbereich 5) of the Goethe-university Frankfurt was obtained prior to the study.

### Participants

At t_1_, the first page of the online assessment was opened *N* = 2899 times. Informed consent was provided by *n* = 1545 (53 % of the total sample), and of the starters, *n* = 957 (62 %) participants completed the first assessment. Exclusion criteria included self-reported schizophrenia (meaning that the participants chose one of the following diagnoses: Schizophrenia, schizotypal and delusional disorders or “psychosis”) and a time taken to complete the survey of less than 10 min (8 min at t_2_ because the participants were already familiar with the procedure), which we took as an indication of a standardized response pattern. Participants with self-reported schizophrenia were excluded, to reduce the risk of analyzing hallucinatory imagery, in which individuals cannot differentiate between mental image and reality. Other psychopathologies were not excluded, as we aimed to assess mental imagery in a sample with maximum diversity.

Forty-five individuals were excluded post-hoc due to a reported life-time diagnoses of schizophrenia (*n* = 35) or completion times of less than ten minutes (*n* = 10), resulting in a remaining sample of *n* = 912. At t_2_, *n* = 733 participants who agreed to take part in the follow-up assessment (80 % of the completers at t_1_) were contacted via email. Of those, *n* = 572 (78 %; 63 % of the completers at t_1_) began the survey, *n* = 425 (58 %) provided a web-based written informed consent, *n* = 418 (57 %) entered their code, and *n* = 352 (48 %; 39 % of the completers at t_1_) completed all of the questionnaires. One hundred and seventy eight (51 %) participants were screened out due to incomplete or non-relatable codes (*n* = 163); e.g. a personalized code was generated by the participant at t_1_ and matched to his or her dataset, but the participant entered an incomplete or incorrect code at t_2,_ thus datasets for both time points could not be connected; or due to self-reported schizophrenia (*n* = 13). Two participants were excluded due to time issues, leaving a final sample size of *n* = 174 (19 % of the completers at t_1_). Table 1 provides an overview of the sample characteristics of the participants who completed the study.

**Table 1:** Sample characteristics of completers

Characteristics	t_1_ (*n* = 912)	t_2_ (*n* = 174)
Age^a^	29.7 (10.2)	30.1 (9.8)
Gender (female)^b^	71.4	76.4
Time to complete^c^		
Mean	55.9 (109.7)	40.5 (22.7)
Median	39.9	35.8
Highest level of education^b^		
No graduation (yet)	1.7	0.6
Lower secondary education	1.9	0.6
Secondary school	9.9	8.0
High-school diploma	49.1	51.1
Bachelor degree	11.8	11.5
Master degree	18.8	17.8
Doctorate	1.9	1.2
Other	4.9	9.2
BDI-II score^a^	15.2 (13.5)	15.5 (14.3)
Self-reported diagnoses^b^ (multiple answers possible)		
Depression	32.7	44.3
Bipolar disorder/mania	2.1	0
Substance addiction	4.2	2.3
Borderline personality disorder	16.9	19
Social anxiety disorder	9.8	10.9
PTSD	13.8	18.4
none of the above	61.3	51.1

*Note*. Standard deviation (SD) in parentheses if in order

^a^Mean values are displayed

^b^Percentage

^c^In minutes. Only for participants without discontinuity for 30 min or above (*n* = 864 at t_1_ and *n* = 157 at t_2_)

### Materials

#### Depression

To assess depressive symptoms, an online-version of the German Beck Depression Inventory (BDI)-II [34] was used, which can be transferred into a non-paper and pencil format without the loss of psychometric properties [35]. The BDI-II is a well-known and reliable instrument to assess depressive symptoms in clinical and non-clinical populations. The BDI-II was found to have a Cronbach’s α ≥0.84 for non-depressed and acutely depressed samples as well as adequate content validity and sufficient retest-reliability in non-clinical samples [36].

### Positive and negative imagery questionnaire

Its general structure was based on the items used in the Intrusion Interview and the Intrusion Questionnaire by Hackmann and colleagues [37]. It was expanded to include emotions, thoughts and coping strategies in response to imagery and included an additional section on positive mental images and ID-images. Participants were first provided with a description of the different types of mental images. They were then asked whether they had experienced such mental images. Initially, general items regarding the frequency of positive, negative or ID-images were presented, followed by a section on the most significant image. Participants were then asked to provide a detailed description of the image (instructions: “Please describe your […] image as if you were explaining a picture or a film clip to someone who is unable to see it.”), and all of the subsequent items referred to this specific image during the past two weeks. Imagery characteristics like vividness, controllability (perceived controllability or uncontrollability was used as a measure of intrusiveness), nowness (the extent to which what was happening before one’s mind’s eyes felt like it happened here and now, as compared to being a “thing from the past”), distress, and pleasantness were rated on visual analogue scales ranging from 0 (“not at all”) to 100 (“extremely”). The item “mood change in response to imagery” was also rated on a visual analogue scale from 0 to 100, with 50 as an anchor for “no mood change” (e.g. participants felt that the occurrence of the specific image had no influence on their mood at the time). Values below 50 therefore indicated a worsening of mood as compared to the time before the image occurred, and above 50 an improvement of mood due to the image. Results on qualitative imagery characteristics refer to the central image, not the total of all images experienced by the participant. For the analyses, a subset of items that were relevant to our hypotheses was used.

### Self-reported diagnoses

Participants were asked to indicate whether they had been previously diagnosed with any of the following mental disorders: Schizophrenia, bipolar disorder or mania, alcohol or drug addiction, depression, borderline personality disorder (BPD), social phobia, or PTSD.

### Additional instruments

Measures also included the short version of the Borderline Symptom List (BSL-23 [38]), the Posttraumatic Diagnostic Scale (PDS) to assess the severity of posttraumatic stress symptoms [39], and a short instrument for social anxiety (Mini-Social Phobia Inventory [40]). Due to the focus on depressive symptoms and our research questions, data from these questionnaires will not be reported in this article.

### Procedure and recruitment

To reach a sample with sufficient variance in depression, participants were recruited using social media websites, e-mail distribution lists, online forums for mental disorders, and notices on several local bulletin boards. In addition, flyers were distributed in psychiatric hospitals and outpatient clinics. Here, a description of the study was given, including a hyperlink to the survey homepage, which led to the first page of the assessment. The cover page displayed the study information, the contact address of the first two authors and a declaration of anonymity. Warnings were included when the study was advertised on specific internet forums on mental disorders (“In this study, you will among other things, be asked about negative experiences that you might have had, which can lead to emotional distress. If you know that you might have extreme reactions when faced with such questions or if you feel very distressed during the assessment, we advise you to discontinue your participation or to refrain from starting the survey.”; translation by the first author). Participants were asked to provide informed consent. The mental image questionnaire included filter rules, which meant that specific areas were presented only if the person reported experiencing the type of imagery in question.

Recruitment took place for a period of four months for the first assessment. Participants were then asked for permission to be contacted for a second assessment 8 weeks after their initial participation. Participants which agreed to participate in the follow-up assessment were sent an email with a link to the second assessment two month after their first participation. As an incentive for participation in the study, three iPads were raffled off for the completion of both assessments.

### Statistical analyses

Spearman correlations were calculated to determine correlations between depression severity and imagery characteristics. To assess the differences between the characteristics of certain imagery types, repeated-measures analyses of variance were conducted. T-tests were used when comparing groups of participants divided into those with and without certain image-types, when comparing depression severity with relation to self-reported diagnoses and when contrasting depression scores in participants with and without mood-worsening after mental imagery. To examine relationship between mental imagery and depression at the cross-sectional and longitudinal levels, path analysis models were specified and evaluated. These models can be used to simultaneously compute regression analyses for multiple independent and dependent variables [41]. Thus, direct and indirect relationships between variables can be assessed. Depression scores at both measurements were used as the primary dependent variables. As predictors, imagery characteristics, which have been widely studied in the literature to date – namely, frequency, vividness, controllability, and nowness – were assessed to determine their contribution to depression severity.

### Data preparation

Prior to data analysis, participants were screened based on the previously outlined exclusion criteria, and the data sets for t_1_ and t_2_ were checked for completeness and correctness. For the longitudinal analyses, data from both measures were matched based on personalized codes, and inconsistent cases (e.g., incomplete codes; duplicates from the same IP-address; no repeated participation) were manually excluded. Because all of the responses were ‘forced-choice,’ no missing data were generated. For all of the analyses of t_1_ only, the complete sample was used. Models regarding comparisons between both time points excluded those subjects at baseline who did not participate at t_2_. When not otherwise specified, the results refer to the sample at t_1_.

### Path analysis models

As described previously, two models (Model 1 and Model 2) with three independent variables (the total number of positive, negative, and ID-images) were computed. Model 1 contained one dependent variable (depression at t_1_), and Model 2 contained two dependent variables (depression at t_1_ and t_2_). Two models (Model 3 and Model 4) with five independent variables (frequency - number of days within a two week period, frequency per day, vividness, controllability and nowness) were computed. Model 3 contained two dependent variables (imagery distress at t_1_ and depression at t_1_), and Model 4 contained three dependent variables (imagery distress at t_1_, depression at t_1_, and depression at t_2_).

### Model estimation and evaluation of model fit

The four path analysis models were analyzed using M*plus*, version 6 [42] by applying the maximum likelihood estimator with robust standard errors (MLR) and the chi-square test statistic, both of which are robust in regard to non-normality, to account for some of the variables displaying significant deviations from the normal distribution. For model fit evaluation, the Satorra-Bentler *χ*^2^ -value (SBχ^2^) and degrees of freedom (*df*) were reported, as well as the root mean square error of approximation (RMSEA), the comparative fit index (CFI), and the standardized root mean square residual (SRMR). There are certain standards governing model fit [43]. Specifically, values below 2 for the ratio between the *χ*^2^-value and *df* indicate a good model fit, and values below 3 indicate an acceptable model fit. RMSEA values less than .05 were found to indicate a good model fit, and values less than .08 indicated an acceptable model fit. The CFI indicates a good model fit for values ranging from .95 to 1.00, whereas values ranging from .90 to .95 signify an acceptable fit. SRMR values should remain below .10 for an acceptable model fit.

## Results

### Descriptive psychopathology data

#### Depression

At t_1_, the mean BDI-II score was 15.2 (*SD* = 13.5). Data ranged from 0 to 58, with a median value of 10. When considering cut-offs for symptom severity, 42.3 % of the sample did not display any relevant symptoms (0–8), 17.4 % scored within the category of minimal depression (9–13), 10.2 % scored within the category of mild depression (14–19), 11.2 % scored within the category of moderate depression (20–28), and 18.9 % were severely depressed (29–63) according to BDI-II cut-offs [34]. Participants with a self-reported lifetime diagnosis of depression had significantly higher BDI-II scores (*M* = 24.9, *SD* = 14.4) than those without this diagnosis (*M* = 10.4, *SD* = 9.9; *t*(439) = −15.72, *p* < .001, *d* = 1.26).

### Prevalence and frequency of mental images

For an overview of imagery characteristics, as well as their correlation with the depression score, see Table 2. Positive mental images were generally experienced by 86.5 % (*n* = 789) of participants, while 77.3 % (*n* = 705) reported experiencing negative mental images, and 34.2 % (*n* = 312) reported experiencing ID-images (for example: “I am sitting on the floor and slit my own wrist.”, “I injure myself by cutting with a scalpel at an intensity that has not happened in real life so far. I can identify the different layers of skin, muscles, tendons, and bones, and I feel good while doing it. The flowing blood calms me, and I am completely relaxed. The consequences of my behavior are irrelevant to me, even if they lead to my death.”, “Friends and family discover my body after my suicide and blame themselves, that nobody was there for me before.”; translation by the first author). T-tests revealed significantly higher depression scores in participants not reporting any positive images (*M* = 25.7, *SD* = 16.1) than in those who did report these images (*M* = 13.5, *SD* = 12.3; *t*(144.8) = 7.9, *p* < .001, *d* = 0.95). Higher depression scores were also identified in participants experiencing negative imagery (*M* = 17, *SD* = 13.9) than in participants who were not experiencing negative imagery (*M* = 8.9, *SD* = 9.4; *t*(500.7) = −9.8, *p* < .001, *d* = 0.62). The same was true for depression severity and the presence (*M* = 22.1, *SD* = 14.8) vs. absence of ID-images (*M* = 11.5, *SD* = 11.2; *t*(500.5) = −11.1, *p* < .001, *d* = 0.85).

**Table 2:** Characteristics of Mental Images at t_1_

Characteristics	Positive images^1^		Negative images^2^		ID images^3^		Correlations with depression score^1,2,3^
	*M*	*SD*	*M*	*SD*	*M*	*SD*	
Prevalence^a^	86.5		77.3		34.2		/
Frequency/number of days in 2 weeks	4.5	(4.7)	3.4	(4.5)	1.2	(2.9)	*r* = −.25^1***^, *r* = .45^2***^,*r* = .44^3***^
Frequency per day	1.8	(3.3)	1.7	(3.7)	0.6	(2)	*r* = −.05 ^*n.s*.^, *r* = .27^2***^, *r* = .36^3***^
Number of different images^b^	3.8	(3.6)	3.3	(3.5)	0.9	(1.8)	*r* = −.26^1***^, *r* = .33^2***^, *r* = .36^3***^
Pleasantness Distress	72.4/	(23.3)/	/43.5	/(28.5)	40.1/	(26.1)/	*r* = −.37^1***^, *r* = .55^2***^, *r* = −.07^3 *n.s*.^
Controllability	72.9	(28.4)	31.9	(28.6)	53.6	(32.5)	*r* = −.23^1***^, *r* = −.29 ^2***^, *r* = −.26^3***^
Nowness	50.7	(31.2)	54.8	(32.2)	49.8	(30.4)	*r* = −.08^1*^, *r* = .32^2***^, *r* = .04^3 *n.s*.^
Vividness	70.9	(24.8)	66.9	(27.4)	62.7	(26.9)	*r* = −.05^1 *n.s*.^, *r* = .30^2***^, *r* = .13^3*^

*p < .000***, p < .001**, p < .05*

*Note*. ^a^Percentage

^b^Sum score

On average, in the present sample at t_1_, positive mental images occurred on 4.5 days during the two weeks preceding the assessment, and on those 4.5 days, the images occurred 1.8 times. Negative mental images occurred less frequently than positive ones, with a mean occurrence of 1.7 times on 3.4 days within the preceding two weeks. ID-images were the least frequent, occurring 0.6 times per day on 1.2 days per week.

Significant positive correlations were found between depression and the frequency (number of days in two weeks) of negative and ID-images, and a significant negative correlation between depression and the frequency of positive images was also found. Regarding the frequency per day with which these images occurred, negative and ID-images were substantially correlated with depression scores, but positive images were not.

### Quantity and proportion of the types of mental images

Regarding the overall number of different images, only negligible differences were detected when comparing positive (*M* = 3.8, *SD* = 3.6) and negative (*M* = 3.3, *SD* = 3.5) mental images, *F*(1, 911) = 9.315, *p* < .05. The Analysis of Variance (ANOVA) revealed a significantly lower number of ID-images (*M* = 0.9, *SD* = 1.8) when compared to both positive (*M* = 3.8), *F*(1, 911) = 495.35, *p* < .001, η^2^ = .35 and negative mental images (*M* = 3.3), *F*(1, 911) = 473.18, *p* < .001, η^2^ = .34. Correlational analyses revealed that higher depression scores were related to a higher number of different negative and ID-images and to a lower number of different positive mental images.

The majority of the participants reported in general both positive as well as negative images (*n* = 619, 67.9 %), 18.6 % reported positive images only (*n* = 170), 9.4 % negative images only (*n* = 86) and the minority (*n* = 37, 4.1 %) reported neither positive not negative images. The highest depression scores were found in the “negative only” group (*M* = 30.6, *SD* = 14.8), which were twice as high as in the group of participants with “both positive and negative images” (*M* = 15.1, *SD* = 12.7) or “neither positive nor negative images” (*M* = 14.3, *SD* = 13.2). Those “positive only” group was found to have the lowest depressions score (*M* = 7.7, *SD* = 7.8). The ANOVA with post-hoc contrasts revealed highly significant differences between all subgroups (*p* < .05) except for the comparison between both imagery types present and none of the imagery types present (*p* = .71).

The proportion of the number of positive images to the number of negative images was negatively correlated with the BDI-II (*r* = −.49, *p* < .001) – the higher the depression score, the greater the extent to which the number of negative images outweighed the number of positive images.

### Imagery characteristics

#### Distress and pleasantness

Mean distress ratings for the most prominent negative images during the preceding two weeks were moderate (*M* = 43.5, *SD* = 28.5; scale ranging from 0 to 100 for all of the images and characteristics). The BDI-II total score correlated positively with imagery distress (see Table 2). Positive images were perceived as mostly pleasant (*M* = 72.4, *SD* = 23.3), and their perceived pleasantness was negatively correlated with depression severity. Despite their objectively negative content, ID-images were also described as moderately pleasant (*M* = 40.1, *SD* = 26.1), but no significant association was found between these images and the depression score.

### Vividness and nowness

Vividness ratings were highest for positive images (*M* = 70.9, *SD* = 24.8), followed by negative images (*M* = 66.9, *SD* = 27.4) and ID-images (*M* = 62.7, *SD* = 26.9). A small significant correlation was found between the vividness of negative images and depression severity. ID-vividness was only marginally correlated with the BDI-II score, and no connection was found between the vividness of positive images and depression.

The perceived feeling of nowness was highest for negative images (*M* = 54.8, *SD* = 32.2), followed by positive images (*M* = 50.7, *SD* = 31.2) and ID-images (*M* = 49.8, *SD* = 30.4). Higher depression scores were correlated with higher levels of perceived nowness of negative images and with lower levels of nowness of positive images. The nowness of ID-images was not associated with depression.

### Controllability

On a scale ranging from 0 to 100, positive images were perceived as mostly controllable (*M* = 72.9, *SD* = 28.4) and negative images as relatively uncontrollable (*M* = 31.9, *SD* = 28.6). ID-images were perceived as more controllable than negative images (*M* = 53.6, *SD* = 32.5). Significant, but small, negative correlations were found between depression and the controllability of positive, negative, and ID-images. Higher depression scores were generally associated with reduced mental image controllability.

### Mood change in response to imagery

In general, participants reported a mood improvement in association with positive imagery (*M* = 72.7, *SD* = 25.4), with a value of 50 indicating no change in mood, values lower than 50 indicating mood worsening and values greater than 50 indicating mood improvement. In contrast, negative images were associated with a deterioration in mood (*M* = 17.5, *SD* = 16.6). Of those experiencing a positive image in the past two weeks (*n* = 757), 17 % (*n* = 129) reported mood deterioration as a result of that image. Participants describing no mood change or mood improvement (*n* = 628) had significantly lower depression scores (*M* = 11.4, *SD* = 11.1) than those who said that their mood worsened after positive imagery (*M* = 22, *SD* = 13.6; *t*(164.6) = −8.34, *p* < .001, *d* = 0.92). Baseline BDI-II scores as well as positive imagery variables (controllability, nowness, and vividness) were simultaneously entered into a regression model predicting mood change. In the “no deterioration”-group, all independent variables obtained comparable β-values of between .12 (vividness) and -.16 (depression severity). All imagery characteristics were significant predictors of higher mood following the mental image. The model achieved an adjusted *R*^2^ of .10. In contrast, in the “deterioration”-group (adjusted *R*^2^ of .20), depression severity was the single most important predictor, with a standardized β-value of -.39. In this group, the contribution of nowness was no longer significant (β = .07, *p* = .42) and vividness yielded a negative β-value (−.18, *p* < .05). Overall, mood remained relatively stable following ID-images (*M* = 45.9), but data was characterized by a large variation between the participants (*SD* = 25.3). Higher depression scores were associated with a lesser improvement in mood following positive images (*r* = −.39, *p* < .001) as well as a greater deterioration of mood following negative images (*r* = −.38, *p* < .001). In contrast, no correlation was found between depression severity and mood change after ID-images (*r* = −.09, *p* > .05), with a modal value of 49. Due to the surprising lack of an association between ID-imagery, depression and mood change, further analyses were carried out. Higher controllability (*r* = .41, *p* < .001) as well as higher levels of pleasantness (*r* = .60, *p* < .001) of ID-images were significantly correlated with better mood following the image, although both variables showed only a small interrelation (*r* = .18, *p* < .05).

### Cross-sectional and longitudinal path analysis models

**Fig. 1:**
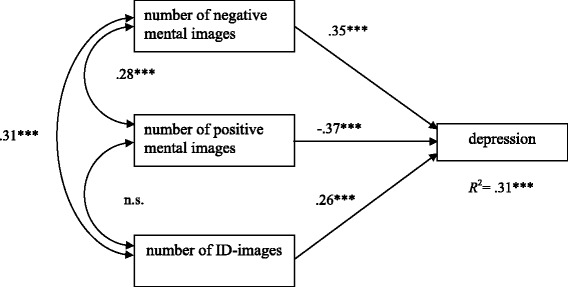
Cross-sectional model at t_1_. Completely standardized solution of a path analysis model with the three independent variables „number of negative mental images“, „number of positive mental images “, and „number of ID-images “and depression severity as the dependent variable. *p < .05, **p < .01, ***p < .001

**Fig. 2:**
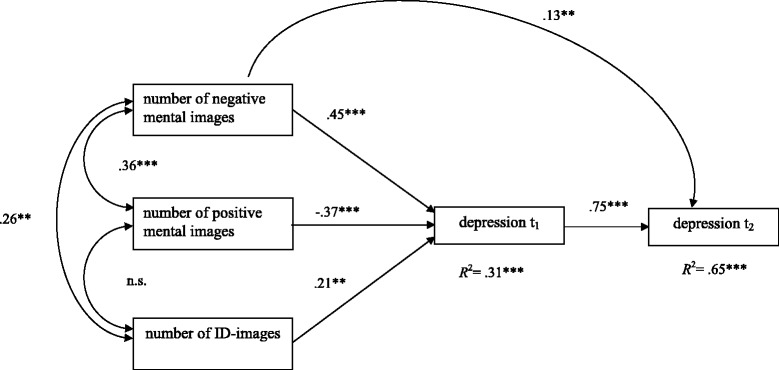
Longitudinal model t_1_ – t_2_. Completely standardized solution of a path analysis model with the three independent variables "number of negative mental images", "number of positive mental images" , and "number of ID-images“and two dependent variables (“depression t_1_” and “depression t_2_"). ***p < .001

To assess the coinstantaneous relationship of positive, negative and ID-images with depression severity at baseline (hypothesis 1) and at the follow-up (hypothesis 2), cross-sectional and longitudinal path analysis models were conducted. Figure 1 shows the cross-sectional path analysis model (Model 1) at t_1_ that relates to the influence of the number of mental images on depression severity. Model 1, with 912 participants, displayed good model fit (SBχ^2^ = 0.49, *df* =1, RMSEA = .0, CFI = 1.00, SRMR = .01). The total *R*^2^ of depression severity at t_1_ reached .31 when these basic imagery variables were included. The longitudinal model (Model 2) extends Model 1 by including depression severity at t_2_ (Fig. 2). The 174 participants with complete data sets for both time points were included. Model 2 also displayed good model fit (SBχ^2^-value: 3.14, *df* =3; RMSEA = .02, CFI = .99, SRMR = .0.03). The *R*^2^ for depression at t_1_ remained unchanged. For depression at t_2_, the *R*^2^ was .65 when imagery characteristics and prior depression score were used as predictors. As indicated, depression severity remained relatively stable in this subsample. In addition to baseline depression, the number of negative mental images significantly contributed to the prediction of depression severity two months later. In the two subsequent models, cross-sectional and longitudinal designs were computed.

**Fig. 3:**
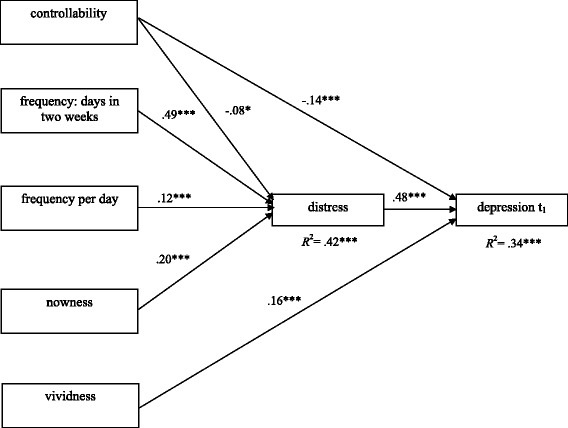
Cross-sectional model at t_1_. Completely standardized solution of a path analysis model with the five independent variables „controllability“, „frequency: days in two weeks “, and „frequency per day “, “nowness” and “vividness” of negative mental images. Two dependent variables have been defined: “distress” and “depression t_1_”. For reasons of clarity and comprehensibility, both relationships between the independent variables and non-significant relationships between independent and dependent variables are not displayed. **p < .01, ***p < .001

In Model 3 (cross-sectional, hypothesis 3), only participants suffering from negative mental images during the preceding two weeks were included in the analysis, leading to a sample of *n* = 550 (see Fig. 3). Model 3 displayed good model fit (SBχ^2^-value: 6.50, *df* =4; RMSEA = .03, CFI = .99, SRMR = .0.01). The *R*^2^ for depression at t_1_ was comparable to that of the previous models (.34). Perceived controllability, the number of days during the preceding two weeks when the most prominent negative image occurred, the intrusion frequency per day, and the nowness and vividness of the image were included as independent variables. Image distress and depression severity functioned as dependent variables. Vividness did not contribute significantly to image distress, yet it was a meaningful predictor of depression severity. In contrast, perceived controllability did contribute to both variables, and imagery distress was the most important contributor to depression. The remaining independent variables contributed to depression severity indirectly via their influence on image distress, which may, therefore, be seen as a mediating factor.

**Fig. 4:**
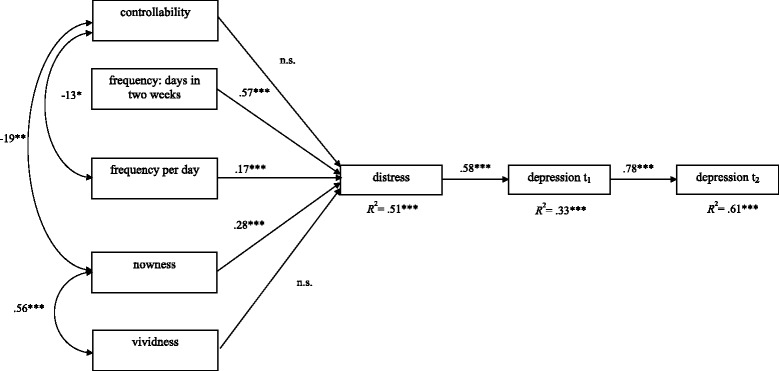
Longitudinal model t_1_ – t_2_. Completely standardized solution of a path analysis model with the three independent variables „frequency: days in two weeks“, „frequency per day “, and “nowness”. Three dependent variables have been defined: “distress” and “depression t_1_” and “depression t_2_”. *p < .05, ***p < .001

Model 4 (longitudinal, Fig. 4, hypothesis 4), with 117 data sets included, also displayed good model fit (SBχ^2^-value: 25.4, *df* =18; RMSEA = .06, CFI = .98, SRMR = 0.08). As was the case in Model 3, only those experiencing negative images during the preceding two weeks were included. The *R*^2^ for depression was .33 at t_1_ and was .61 at t_2_. Contrary to Model 3, controllability and vividness were only indirectly linked to image distress and depression severity through their connection to the other predictors. For this reason, relationships between these two variables and the remainder of the independent variables are displayed.

The number of mental images alone explained approximately one-third of the depression variance. When including more detailed imagery characteristics, such as the frequency or nowness of negative images, similar results were yielded. These variables contribute significantly to imagery distress, which, in turn, is strongly associated with depression severity. The number of negative mental images is a relevant predictor of depression scores two months later, even when accounting for baseline depression in the path model.

### The differential influence of positive and negative mental images

To examine possible differences in the influence of positive and negative images, imagery distress was first entered into a multiple regression model that predicted depression scores at t_1_. In a second step, imagery pleasantness was added as a predictor.

Both independent variables (pleasantness: *p* < .001; distress: *p* < .001) contributed significantly to the prediction of depression (adjusted *R*^2^ step 1 = .31, adjusted *R*^2^ step 2 = .39), with a significant *F*-change from step 1 to step 2. For image distress following negative images, the β-value was .56 at step 1 and decreased to .49 at step 2. For imagery pleasantness following positive images, the β-value was -.29 at step 2. The pleasantness of positive images therefore (negatively) contributes to depression severity, even when the distress caused by negative images is taken into account.

## Discussion

In the present study, we aimed to assess the mechanisms underlying the relationship between mental images and depressive symptoms. We extended research beyond negative imagery to examine the influence of positive images, as well as images of suicide or self-harm, on mood. With a mean of 15.2, the BDI-II score in our sample was lower than those in currently depressed samples [23] but it was comparable to the mean BDI-II scores of formerly depressed participants in other studies [1].

Greater than 77 % of the total sample experienced negative mental images, which is comparable to the prevalence rates of 32 to 96 % of depressed individuals that have been reported in clinical samples [1, 23, 25]. Positive images were also experienced by more than two-thirds of our sample. Interestingly, ID-images were not a rare phenomenon. To our knowledge, this is the first study to assess the prevalence of mental images on injury or death leading to positive emotions in a large sample. Future research on this phenomenon should focus on a comparison of ID-images in different clinical groups or with regard to the presence or absence of psychopathological traits such as anxiety, emotion dysregulation or impulsivity, respectively.

Only small differences were found between the number of positive and negative mental images, but participants reported a significantly lower number of ID-images. Higher numbers of both negative and ID-images as well as a lower number of positive images were related to a greater depression severity. Overall, most participants experienced both positive and negative mental images. A relationship was found between the type of image experienced and self-reported depressive symptoms – individuals who reported only negative mental images also exhibited the highest depression scores (*M* = 30.6), as compared to those who experienced only positive mental images (*M* = 7.7). Interestingly, no differences were found regarding depression severity in those participants who reported either both imagery types or neither of them. We also found that the positive images to negative images ratio was significantly correlated with BDI-II scores: The greater the extent to which the number of negative images outweighed the number of positive images, the higher the depression scores were.

Imagery distress was moderate (43.5 out of 100) – it was lower than that reported by Patel et al. [23], but was higher than the reported distress caused by intrusive images in the study conducted by Birrer et al. [2]. A significant correlation was found between imagery distress and higher depression scores. In contrast, positive imagery pleasantness was negatively correlated with the BDI-II score. ID-images were also described as mostly pleasant, despite their negative content (*M* = 72.4) but ID-pleasantness was not related to depression severity in our sample. The perceived pleasantness of ID-images, combined with their relatively high controllability, may be interpreted as a sign that these images are often intentionally produced rather than intrusive, and they may even be used as a cognitive escape strategy. This idea is in line with the results of Holmes and colleagues [10], with regard to the distress and pleasantness of “flash-forwards” to suicide and their possible role in depression. Keeping in mind the close connection to actual suicidal behaviors that images such as those in the Holmes et al. study [10] have, the high prevalence of ID-images in the present sample is alarming. Further research is required to determine whether there is an increase in the risk of suicide or self-harm due to mental imagery that occurs separately from other psychopathological factors. We believe the mechanisms underlying ID-images are of high priority with regard to both further investigation and the development of interventions for suicidality – both diagnostically, in terms of suicidal risk (and depression severity, in general), and as a crucial target of specialized interventions. At the same time, it is surprising that ID-characteristics such as pleasantness or vividness showed little to no connection to depression severity in the current study. Explanations for the lack of an apparent relationship may be that ID-images did in general occur less frequently than other imagery types and might thus be perceived as less detrimental – possibly through high levels of controllability combined with lower levels of vividness. Further research, especially in clinically depressed patients, is necessary to determine, in which way ID-images differ from other forms of mental imagery to shed light on the role ID-imagery in depression.

Small correlations were found between the vividness and nowness of negative images and depression severity, while others have found nowness to be a key predictor of depression severity [28]. Interestingly, no correlations were found regarding depressive symptoms and the vividness of positive imagery, in contrast to other studies which have found a strong relationship between positive image vividness and depression [24], with a focus on poorer ability to generate vivid positive prospective mental imagery [44]. Explanations for these varying results may be that we did not specifically ask only about future-related positive imagery and asked the participants retrospectively about the vividness of their idiosyncratic positive images instead of having them generate standardized positive scenarios. Those individuals who did not spontaneously report positive images and were therefore excluded in our analysis might be the ones generating not very vivid images in a laboratory task – including only positive images which have occurred in the past restricts variance in vividness analyses. Regarding controllability, higher depression scores were in general associated with low levels of perceived controllability. Negative images were least controllable, as opposed to positive images which were perceived as mostly controllable. ID-images were also moderately controllable.

Of the individuals who reported positive imagery, 17 % experienced mood deterioration following a positive image. These participants also displayed significantly higher depression scores. In the “deterioration”-group, depression severity was the most meaningful predictor, and in contrast to the “no deterioration”-group, higher vividness scores predicted worse mood. Our results imply that mood repair through positive imagery is not self-evident for all individuals – in the worst case, the induction of positive imagery may lead to mood deterioration in individuals suffering from depression [32]. Reasons for this paradoxical effect could be differences in the way in which the images are being processed– an abstract, rumination-focused style versus a more concrete, perception driven mode of thinking [45], as it has been found that (currently and formerly) depressed individuals tend to engage in dysfunctional response mechanisms to positive affect [46]. A possible mood deterioration effect needs be taken into consideration in the treatment of depression when working with positive imagery, for example by instructing patients to use an imagery processing style rather than a verbal, ruminative style [47]. The preliminary results from our study, that participants with a mood deterioration had a depression score twice as high as those without a reported mood worsening through naturalistically occurring positive imagery, need to be investigated further to answer the question whether more severely depressed patients suffer from said mood deterioration in non-laboratory settings. Vividness of positive imagery seems to be an important factor in the mood-repair capabilities of positive images in depression, as it has been found that the effectiveness of symptom reduction in depression via imagery cognitive bias modification (e.g. practicing to generate positive [imagery] resolution to ambiguous stimuli, either auditory or as a picture/word-combination) seems to be dependent upon the level of vividness to which participants can generate positive mental images [48]. Interestingly enough, our results show that positive image vividness could be double-edged sword. As was expected, negative images were overall followed by a worsening of mood, with a greater impact in participants with higher depression scores. Interestingly enough, the majority of individuals did not experience any mood change following ID-images, which was not correlated with depression severity either. The more pleasant and controllable the ID-image was perceived, the better the mood was. It is possible, that participants under-reported very uncontrollable and distressing ID-imagery, which might explain a lack of correlations between mood, depression and these images. Further research should focus on experimental paradigms assessing the causal directionality between ID- images, associated emotion and mood change.

One third of the variance regarding depression severity could be explained by using the number of positive, negative and ID-images as independent variables in the path models. The number of negative images experienced during the same time frame significantly contributed to the prediction of depression severity eight weeks later, over and above baseline depression scores. As postulated, participants who reported experiencing negative images, ID-images, or both were significantly more depressed than those who did not experience these types of mental images. In contrast, the occurrence of positive images was related to lower depression scores. This held true on both the correlational and longitudinal levels, where the presence or absence of these imagery types differentially contributed to depression scores at baseline and follow-up. In addition, when the frequency of negative images was higher, the depression scores were more severe. Further imagery characteristics (controllability, the number of days during the preceding two weeks, intrusion frequency per day, nowness and vividness of the image) were included as independent variables and contributed significantly to imagery distress, which, in turn, was associated with depression severity. Based on the path models, a full mediation of the association between the frequency of mental images and depressive symptoms through imagery distress can be assumed.

Assessing the differential effects of both positive and negative mental images, regression analyses showed that the pleasantness of positive images negatively contributes to depression severity, even when the distress caused by negative images is included.

A major strength of the present study was the use of a large sample composed of greater than 900 participants who displayed a wide range of depression scores and manifestations of imagery. To our knowledge, this is the first mental imagery study containing such a large sample size.

The primary limitations of the present study include the lack of external clinical assessments and the use of a self-selected sample. No statements can be made as to whether participants with high depression scores actually met the criteria for a depressive disorder. Additionally, a history of other diagnoses has also been indorsed by a high percentage of the participants (32.7 % depression, 16.9 % BPD, 13.8 % PTSD, 9.8 % social anxiety disorder, 4.2 substance abuse). The results can thus not be generalized on patients with depression only. However, anonymously conducted internet-based questionnaires can also be considered an advantage of this specific study because are they are associated with lower social anxiety and social desirability scores [49] when compared to the use of non-anonymous paper and pencil formats. Data collection may have facilitated a rather straightforward description of (particularly self-harmful and/or revengeful) mental images, which may have been withheld by participants in a one-on-one setting with a clinician due to these images causing the participants to feel ashamed and to experience other problematic emotions. In order to establish a clear association between persistence or change in depression severity and the presence of mental images and their characteristics, an exhaustive analysis should include testing whether changes in imagery between t_1_ and t_2_ predict changes in depression scores. Moreover, other clinical factors could be influencing depression rates at t_2_. Therefore the longitudinal observations remain preliminary. Additionally, ratings and reports of imagery characteristics were made retrospectively, which could have introduced a memory bias [50]. Future studies could use ecological momentary assessment to address this issue [51]. Self-selection regarding the start as well as the completion of the study are likely to be present, with factors ranging from internet use in general, to concentration over the duration of the assessment, symptom severity (intrusions, distress), among others. A possible mechanism to reduce self-selection bias might be the use of randomization to different online-studies after informed consent on multiple-study websites [52]. Due to the reduction in the sample size based on incorrect identification-codes as well as general attrition, the completers at t_2_ may not be a representative sub-sample of t_1_. With 39 % of the completers at t_1_ also completing measures at t_2_, the rate of attrition is comparable to that found in a study on monthly follow-ups of an internet-based depression screening, where 33.8 % completed one or more follow-ups [53]. Studies on web-based research show that high attrition rates are a common problem [54]. Further research should also include general anxiety measures, to control for possible confounding or mediation of the relationship between mental images and depression severity.

## Conclusions

To date, the role of positive images is still underrepresented in important psychological treatments for depression, but mental imagery may be a rich source for the development of new treatment approaches. To sum up, depression severity was higher in those experiencing no or only a small number of positive mental images, which were then also perceived as less pleasant. Vivid and frequently occurring negative mental images were also connected with higher depression scores. Images of injury and death were also found in our sample, and their presence was associated with a greater depression severity. Due to their relationship with depression severity at baseline as well as follow-up, our results indicated that mental imagery might play an important role in the maintenance of depressive symptoms. The extent to which imagery characteristics like frequency, vividness or controllability seem to contribute to emotional distress may be an important mechanism through which mental images influence psychopathology, especially depression. It has to be noted though, that the experiencing of distressing imagery in general does not seem to be the only important factor as individuals with both positive and negative mental images did not differ from those who experience no mental images at all with regard to their self-reported depression severity. Future research should further elaborate on the intraindividual influence of the adverse ratio of positive versus negative mental images in clinically depressed patients.
